# Supplementary material for: Zika Virus Infection Damages the Testes in Pubertal Common Squirrel Monkeys (Saimiri collinsi)
Source: Viruses. 2023 Feb 23;15(3):615. doi: 10.3390/v15030615 (PMC10051343; doi:10.3390/v15030615)

Supplementary Material

Table S1. Mean ± SD of testosterone levels (ng/dl) in the fecal extract of squirrel monkey (*Saimiri collinsi*): pre inoculation, acute phase, and convalescent phase of Zika virus experimental infection. Values after logarithmic transformation.

	**Pre inoculation**	**Acute Phase**	**Convalescent Phase**
**Group**	Day -5 to -1	Day 0 to 9	Day 10 to 21
G1: Uninfected	2,6450 ± 0,2608[Aa]	2,5954 ± 0,2119[Aa]	2,7294 ± 0,2297[Aa]
G2: Infected	2,7110 ± 0,2213[Aa]	2,4857 ± 0,3232[Ab]	2,6620 ± 0,1582[Aa]

[A-B] Different uppercase letters between rows indicate statistical difference between groups in the same stage of infection (p < 0.05).

[a-b] Different lowercase letters between columns indicate statistical difference between stages in the same experimental group (p < 0.05).

Table S2. Mean ± SD of testicular biometry of the squirrel monkey (*Saimiri collinsi*): acute and convalescent phase of Zika virus experimental infection. Values after logarithmic transformation.

		**Acute Phase**	**Convalescent Phase**
	**Group**	**Day 0, 3, 5 e 7**	**Day 10, 14 e 21**
**Testicular Volume (cm^3)**	G1: Uninfected	-0,092 ± 0,154^{Aa}	-0,077 ± 0,069^{Aa}
	G2: Infected	-0,2655 ± 0,0730^{Ba}	-0,3385 ± 0,0294^{Bb}
**Gonadosomatic Index (%)**	G1: Uninfected	-0,845 ± 0,169^{Aa}	-0,822 ± 0,159^{Aa}
	G2: Infected	-1,0074 ± 0,0949^{Ba}	-1,0812 ± 0,0402^{Bb}
**Body Weight (g)**	G1: Uninfected	2,7722 ± 0,0227^{Aa}	2,7640 ± 0,0115^{Aa}
	G2: Infected	2,7614 ± 0,0393^{Aa}	-2,7623 ± 0,0282^{Aa}

$^{A\text{-}B}$ Different uppercase letters between rows indicate statistical difference between groups in the same stage of infection ($p < 0.05$).

$^{a\text{-}b}$ Different lowercase letters between columns indicate statistical difference between stages in the same experimental group ($p < 0.05$).

Figure S1. Group evaluation, daily data: (A) Testosterone, (B) Testicular Volume, (C) Gonadosomatic Index, and (D) Body weight. Mean and min-max values. The infection stages are represented by the periods: pre inoculation (day -5 to -1), acute phase (day 0 to 9), and convalescent phase (day 10 to 21).

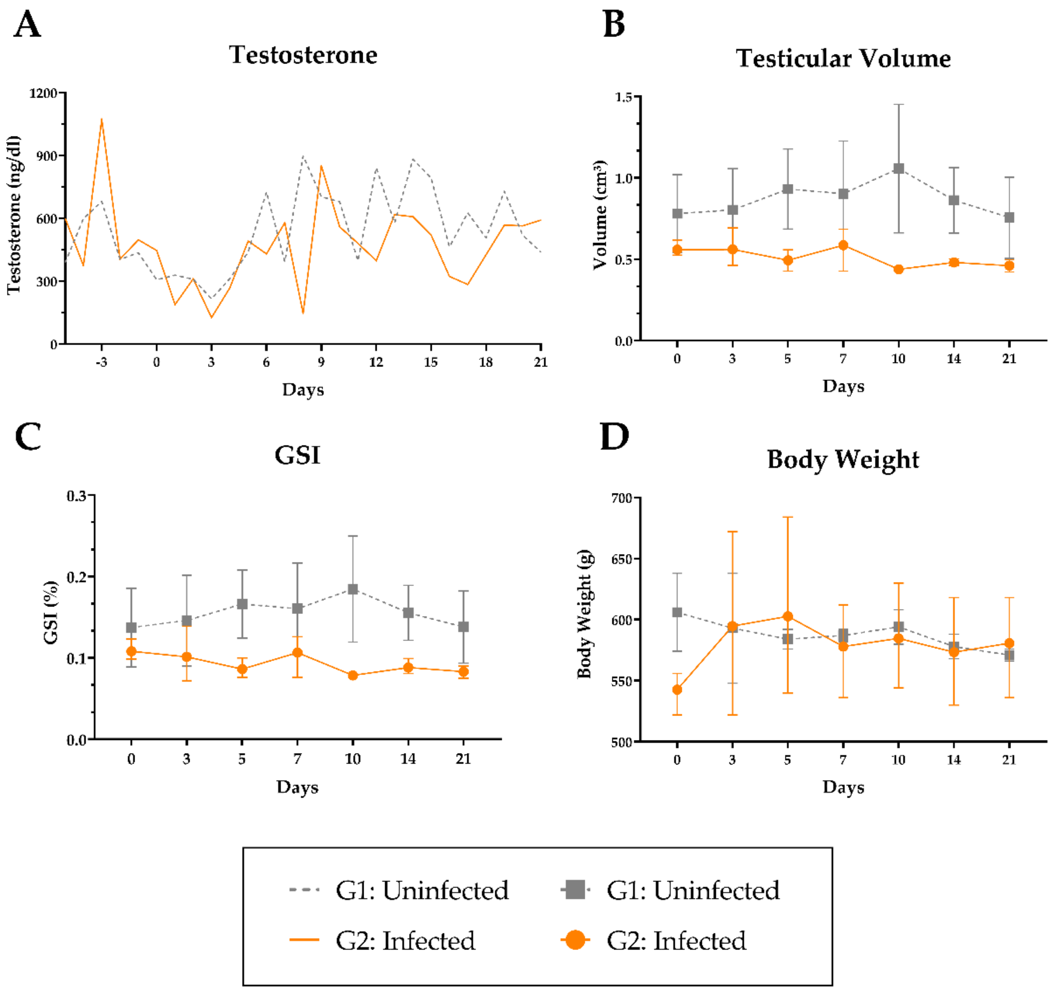

Supplement: Supplementary file 1 [file viruses-15-00615-s001.zip › viruses-1960586-supplementary.pdf]
